# Validation of an adipose-liver human-on-a-chip model of NAFLD for preclinical therapeutic efficacy evaluation

**DOI:** 10.1038/s41598-021-92264-2

**Published:** 2021-06-23

**Authors:** Victoria L. Slaughter, John W. Rumsey, Rachel Boone, Duaa Malik, Yunqing Cai, Narasimhan Narasimhan Sriram, Christopher J. Long, Christopher W. McAleer, Stephen Lambert, Michael L. Shuler, J. J. Hickman

**Affiliations:** 1 NanoScience Technology Center, University of Central Florida, 12424 Research Parkway, Suite 400, Orlando, FL 32826 USA; 2 Hesperos, Inc., 12501 Research Parkway, Suite 100, Orlando, FL 32826 USA; 3 College of Medicine, University of Central Florida, 6850 Lake Nona Blvd, Orlando, FL 32827 USA

**Keywords:** Drug discovery and development, Metabolic disorders, Experimental models of disease

## Abstract

Nonalcoholic fatty liver disease (NAFLD) is the most common liver disease and strongly correlates with the growing incidence of obesity and type II diabetes. We have developed a human-on-a-chip model composed of human hepatocytes and adipose tissue chambers capable of modeling the metabolic factors that contribute to liver disease development and progression, and evaluation of the therapeutic metformin. This model uses a serum-free, recirculating medium tailored to represent different human metabolic conditions over a 14-day period. The system validated the indirect influence of adipocyte physiology on hepatocytes that modeled important aspects of NAFLD progression, including insulin resistant biomarkers, differential adipokine signaling in different media and increased TNF-α-induced steatosis observed only in the two-tissue model. This model provides a simple but unique platform to evaluate aspects of an individual factor’s contribution to NAFLD development and mechanisms as well as evaluate preclinical drug efficacy and reassess human dosing regimens.

## Introduction

Nonalcoholic fatty liver disease (NAFLD), characterized by ectopic lipid storage in hepatocytes not driven by alcohol abuse, is the most common form of liver disease, affecting around 24% of individuals globally^1,2^. While the specific causes of NAFLD are not fully known, it is highly correlated with metabolic syndrome (elevated blood pressure, elevated fasting glucose, and abdominal obesity) and often presents with the comorbidities type II diabetes (T2D) and cardiovascular disease^3^. While NAFLD is typically benign, for 1 in 5 individuals with the disease, NAFLD advances to nonalcoholic steatohepatitis (NASH), which involves liver inflammation and potential fibrosis, with progression to cirrhosis, liver cancer, or liver failure^2^. NASH is the leading cause of liver transplants in women in the US^4^, and with increasing global obesity rates and a growing incidence of metabolic syndrome, it is predicted to be the major cause for liver transplants by 2025^5,6^. However, limited treatment options are available for NAFLD and NASH, with diet and lifestyle modifications typically recommended as the primary intervention strategy^7^. Metformin, a biguanine insulin-sensitizing drug, and other T2D medications have been explored for the treatment of NAFLD, but while some success has been shown with high doses in HepG2 cells^8,9^ and rodent^10^ models, metformin has not been shown to improve liver histology in human clinical trials^11,12^.

Fundamentally, the etiology of NAFLD is not well established; early characterization of NAFLD described pathology brought on by a “first hit” of steatosis, driven by diet and obesity, which initiates a “second hit” of inflammation and fibrosis. It is now thought that liver damage develops as a result of a complex cascade of disease drivers that act in parallel, leading to different phenotypes and levels of progression in all patients^13,14^. When studying NAFLD, it is difficult to separate disease drivers from biomarkers; NAFLD has been associated with changes in cytokine profiles, insulin resistance, and dysfunctional lipid transport, but it is not fully known to what extent these factors initiate damage or are markers of damage. In many cases, inflammation precedes steatosis, and steatosis could be a protective mechanism against dysfunctional lipid metabolism rather than a causative factor^15,16^. As a result, pharmaceutical treatments for NAFLD directed primarily at reducing liver steatosis have limited efficacy, and their targets may only be relevant in certain phenotypes. Further, due to differences in glucose and lipid metabolism between rodents and humans, many NAFLD treatment strategies established in mouse or rat models have not been successful in human trials. Consequently, human based approaches have numerous advantages over animal models, especially for studying metabolic diseases^17^.

NAFLD has been extensively studied in humans using liver biopsy samples. For example, histological studies have been used to identify patterns of liver injury including simple steatosis, hepatocyte ballooning, and Mallory-Denk bodies, and have been used to establish a grading system to characterize disease progression^18^. Further, gene expression studies have revealed changes to genes involved in lipid metabolism, fatty acid transport, and inflammation^19^. However, NAFLD studies in humans are largely correlative, and because of high overlap between NAFLD and other metabolic syndrome diseases, it is difficult to study lipid dysfunction or inflammation in isolation. To address this, NAFLD has been studied extensively in vitro^20–22^, and numerous models for NAFLD and NASH have been established, including microfluidic models^23,24^. However, few models consider the total-body interactions present in vivo. Human-on-a-chip (HoaC) technology is a promising strategy for the study of metabolic disease and NAFLD. Human-based, multi-organ systems, composed of multiple interconnected functional tissue mimics with a serum-free recirculating medium, allow for the study of human cellular interactions in vitro with the potential for disease research and pharmaceutical testing, capable of representing tissues relevant to compound metabolism on a single device^25^. HoaC technology provides a unique opportunity to study metabolic disease in a controlled environment where all components of a microphysiological system are characterized and controlled making it possible to isolate the effects of specific compound or tissue interactions, especially without the compounding effects of serum.

In this study, we developed and characterized a microfluidic HoaC model composed of adipocytes representing white adipose tissue (WAT) and functional liver cell chambers in recirculating, serum-free medium to study aspects of NAFLD and NASH. This system utilizes serum-free media formulations tailored to represent different human metabolic states—healthy, corresponding to normal human postprandial glucose and insulin concentrations, diabetic, with representative glucose and insulin, obese, with representative recirculating free fatty acids (FFA)^20^, and proinflammatory, with tumor necrosis factor-alpha (TNF-α). The gravity-driven platform with recirculating medium allows for non-invasive biomolecule detection assays at any point in a 14-day treatment period, along with functional readouts and endpoint assays, that has shown a significant effect of the adipocyte module on hepatocytes in the NAFLD model, establishing the importance of adipocyte-hepatocyte interactions in establishing a disease model. Further, and most important, we have shown that the anti-steatotic effects of the insulin-sensitizing drugs are only observed at doses well above those clinically administered in humans, demonstrating that this platform can be used to bridge the gap between assays performed in cell lines or rodents and long-term human efficacy, and can be a resource to give insight into why compounds that are successful under some conditions fail in human trials.

## Results

### Characterization of human hepatocytes in monoculture

**Figure 1:**
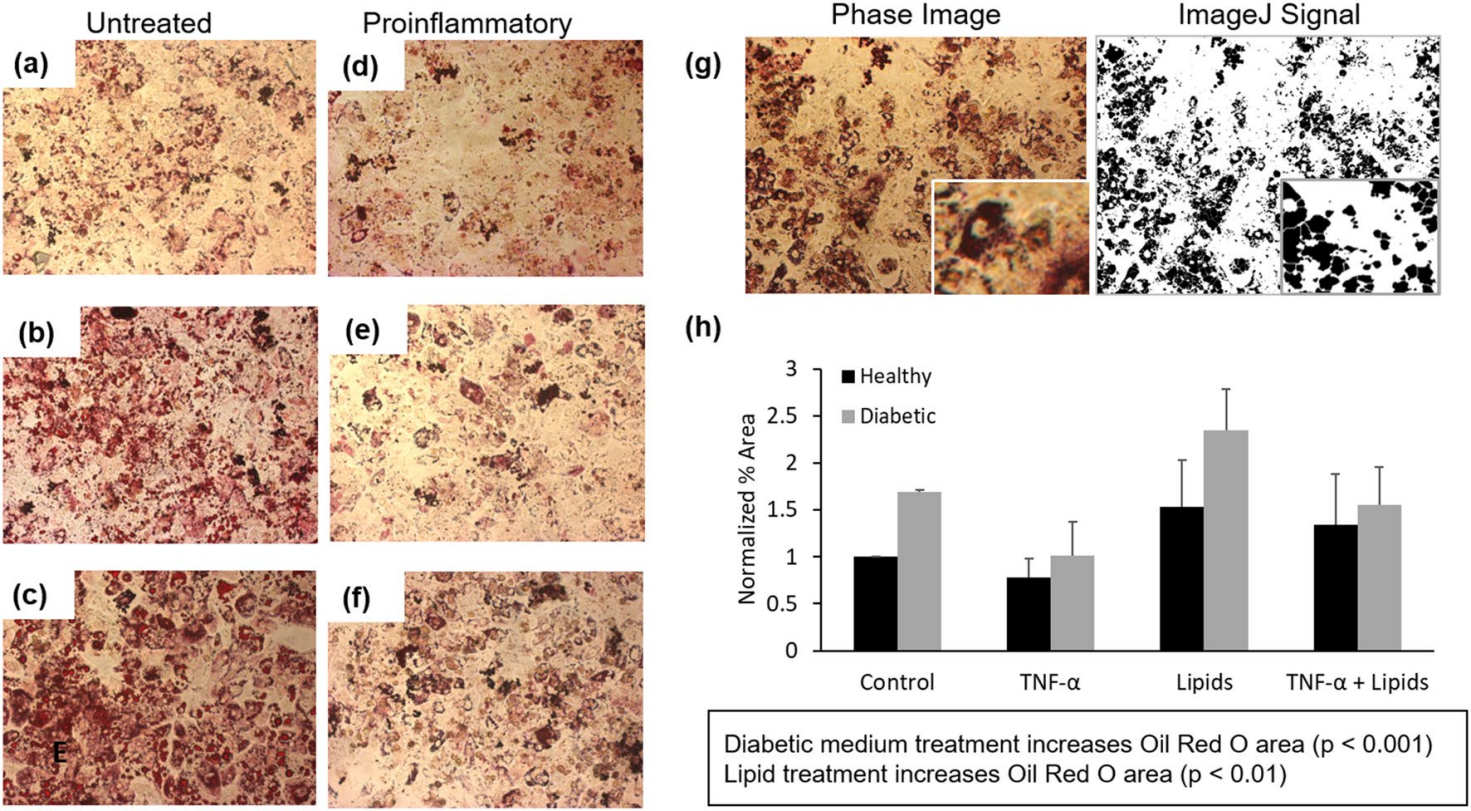
Characterization of hepatocytes in monoculture. Oil Red O staining showing lipid accumulation of hepatocytes after 14 days in (**a**) Healthy BMM (**b**) Diabetic BMM (**c**) Diabetic + obese BMM (**d**) Proinflammatory BMM (**e**) Diabetic + proinflammatory BMM (**f**) Diabetic, obese + proinflammatory BMM). (**g**) Comparison of ORO stained phase image and isolated signal used for quantification. (**h**) Quantification of overall lipid storage using ImageJ analysis. Statistical analysis performed as multi-way ANOVA followed by Dunnett’s test (α = 0.05) using the coverslip-wide average of two independent coverslips per condition.

This study used isolated human hepatocytes obtained from patient samples that were commercially available and de-identified. These human-derived liver cells have been established to maintain viability, CYP1A1 and 3A4 activity, and albumin secretion for up to 28 days in serum-free medium in a single organ platform and in a multi-organ system under flow. The previous characterization of this 2D liver cell module established that the primary hepatocytes maintain a characteristic morphology, express key markers (MRP1, actin and albumin) and produce urea and albumin and maintain CYP P450 activity for 28 days^26^. Further, these cells also maintain their phenotype and CYP 1A1 and 3A4 activity under flow for 28 days in a multi-organ interconnected system^27,28^. Figure 1A–F shows the effects of media combinations designed to simulate different clinical conditions (Methods) on the accumulation of lipids within these cells after 14 days of incubation. Steatosis was visualized with the Oil Red O (ORO) assay. Using ImageJ software, ORO stained phase images were converted to percent area signals (Fig. 1G). As quantified in Fig. 1H, diabetic medium increased steatosis 1.55 times that of the control healthy medium (*p* < 0.001). Lipid treatment also increased steatosis in healthy (1.89×) and diabetic (2.39×) formulations (*p* < 0.01), whereas TNF-α, a cytokine secreted by macrophages in adipose tissue, did not drive increased steatosis in monoculture after treatment (Fig. 1H).

### Characterization of human adipocytes in monoculture

**Figure 2:**
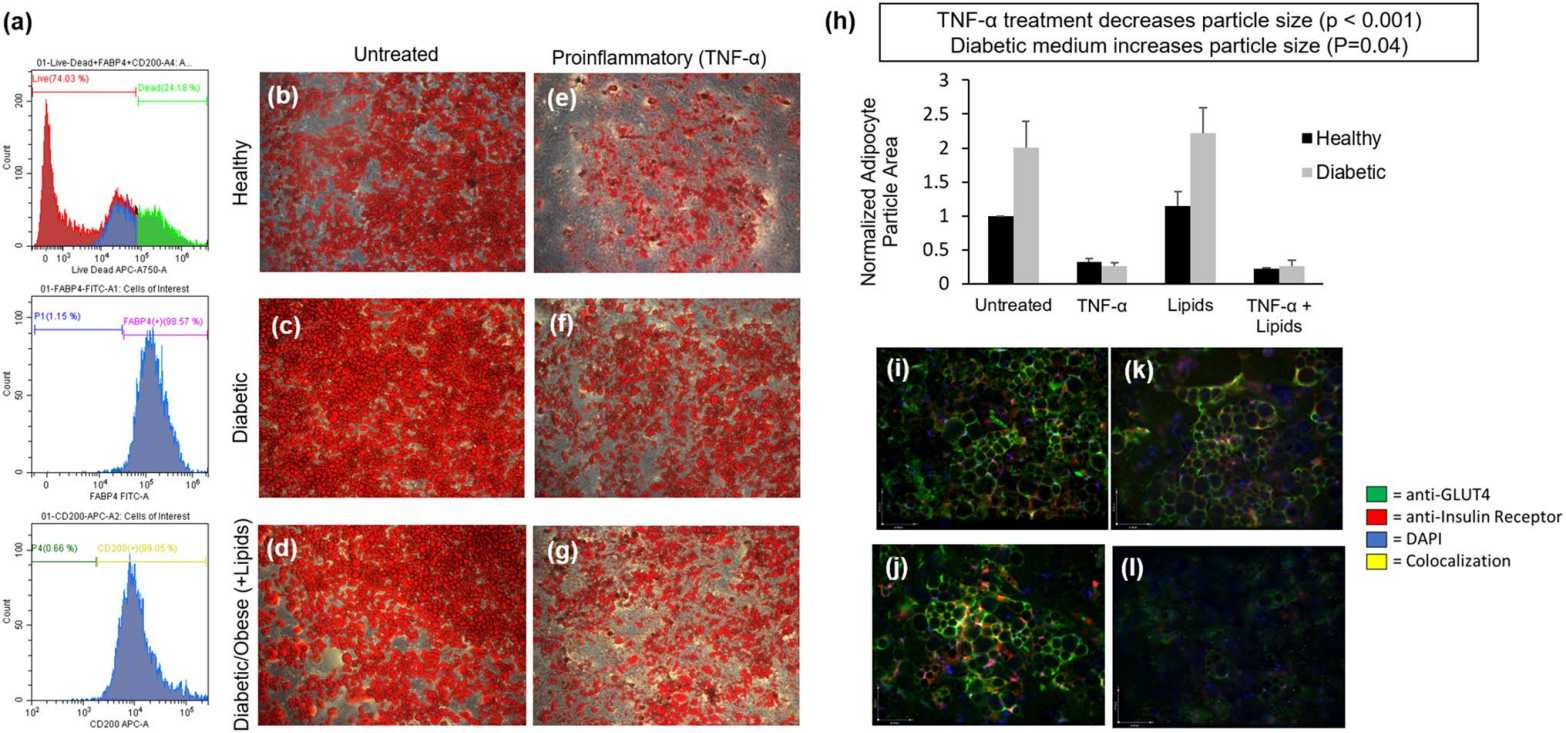
Characterization of adipocytes in monoculture. (**a**) Cytometer live/dead, FABP4, and CD200 staining of adipocytes after 14d differentiation. Oil Red O staining showing lipid storage in adipocytes after differentiation and 14-day treatment in (**b**) Healthy BMM (**c**) Diabetic BMM (**d**) Obese BMM (**e**) Proinflammatory BMM (**f**) Diabetic + proinflammatory BMM (**g**) Obese + proinflammatory BMM). (**h**) Quantification of lipid droplet size using ImageJ particle analysis. ICC quantification of insulin resistance biomarkers after 14d in (**i**) Healthy BMM (**j**) Diabetic BMM (**k**) Diabetic + Obese BMM (**l**) Diabetic, obese, and proinflammatory BMM. Statistical analysis performed as multi-way ANOVA followed by Dunnett’s test (α = 0.05) using the coverslip-wide average of two independent coverslips per condition.

Adipocytes displayed complete differentiation, as shown by the expression of markers CD200, a membrane glycoprotein expressed by visceral adipocytes^29^, and FABP4, a fatty acid binding protein^30^ (Fig. 2A) as well as high levels of consistent lipid storage (Fig. 2B–G). Both diabetic and obese medium formulations led to increases in lipid storage in the adipocytes, though only the diabetic condition exhibited a statistically significant increase (*p* = 0.04) (Fig. 2H). Medium containing TNF-α increased lipolysis in adipose tissue as shown by decreased lipid droplet size in both healthy and diabetic conditions (*p* < 0.001) (Fig. 2H). ICC imaging of monoculture adipocytes indicated evidence of insulin resistance in the proinflammatory medium as shown by a decrease in the expression of GLUT4 and the insulin receptor (Fig. 2I–L).

### Multi-chamber housing

**Figure 3:**
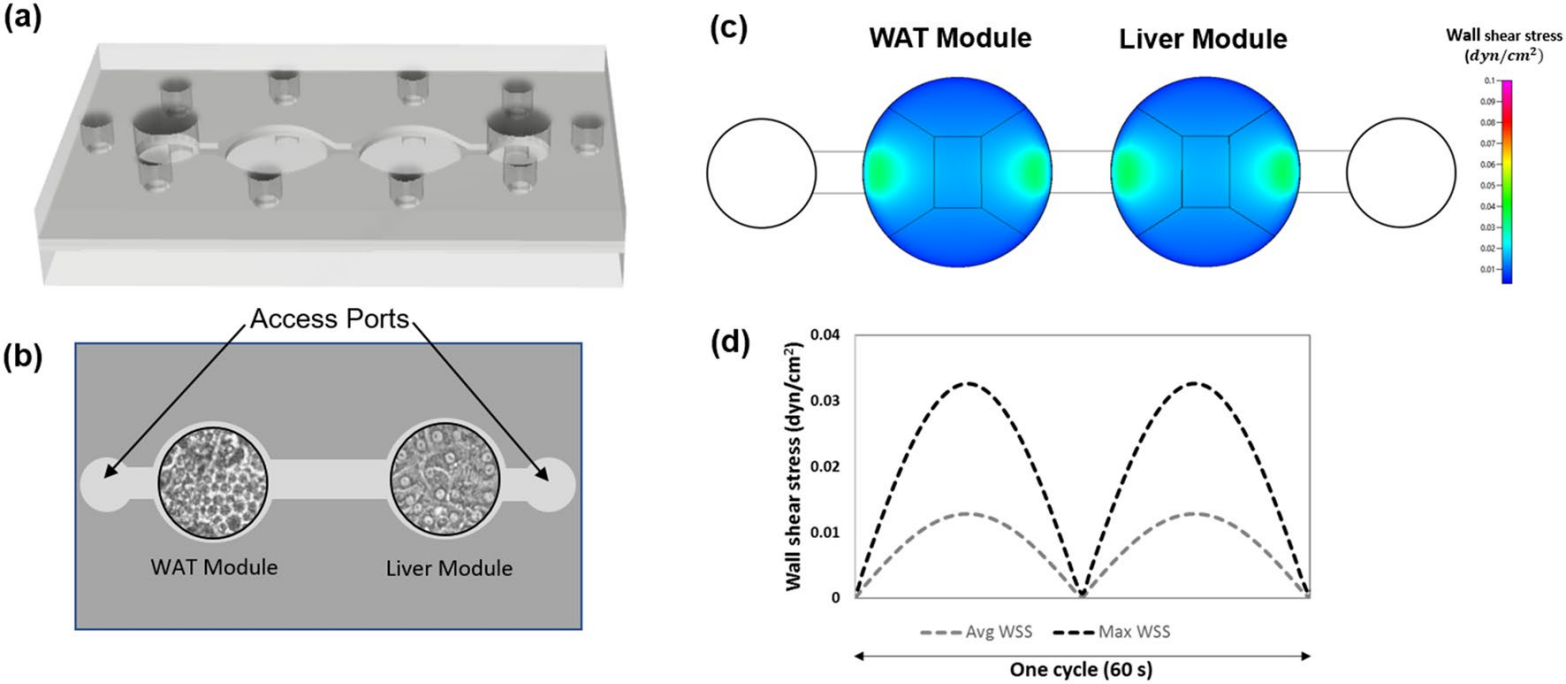
System fabrication and characterization. (**a**) Schematic of 2-chamber microfluidic housing. (**b**) Compartments of 2-chamber housing containing white adipocyte and hepatocyte modules. (**c**) Maximum shear stress distribution. (**d**) Shear stress quantification over one rocker cycle.

The fluid behavior of the medium in the two module housing (Fig. 3) was modeled using CFD for both flow rates and shear stresses. The circular chambers for the two organ tissues demonstrate shear stresses less than 0.05 dynes/cm^2^ at the highest period of flow in the system through the rocking profile time with slight increases in shear stress near the entrance/exit of each chamber (Fig. 3C). This shear stress follows a cyclic behavior due to the oscillating nature of the rocking platform which produces recirculating flow (Fig. 3D).

### Proinflammatory medium increases hepatocyte lipid storage in a multi-chamber platform

**Figure 4:**
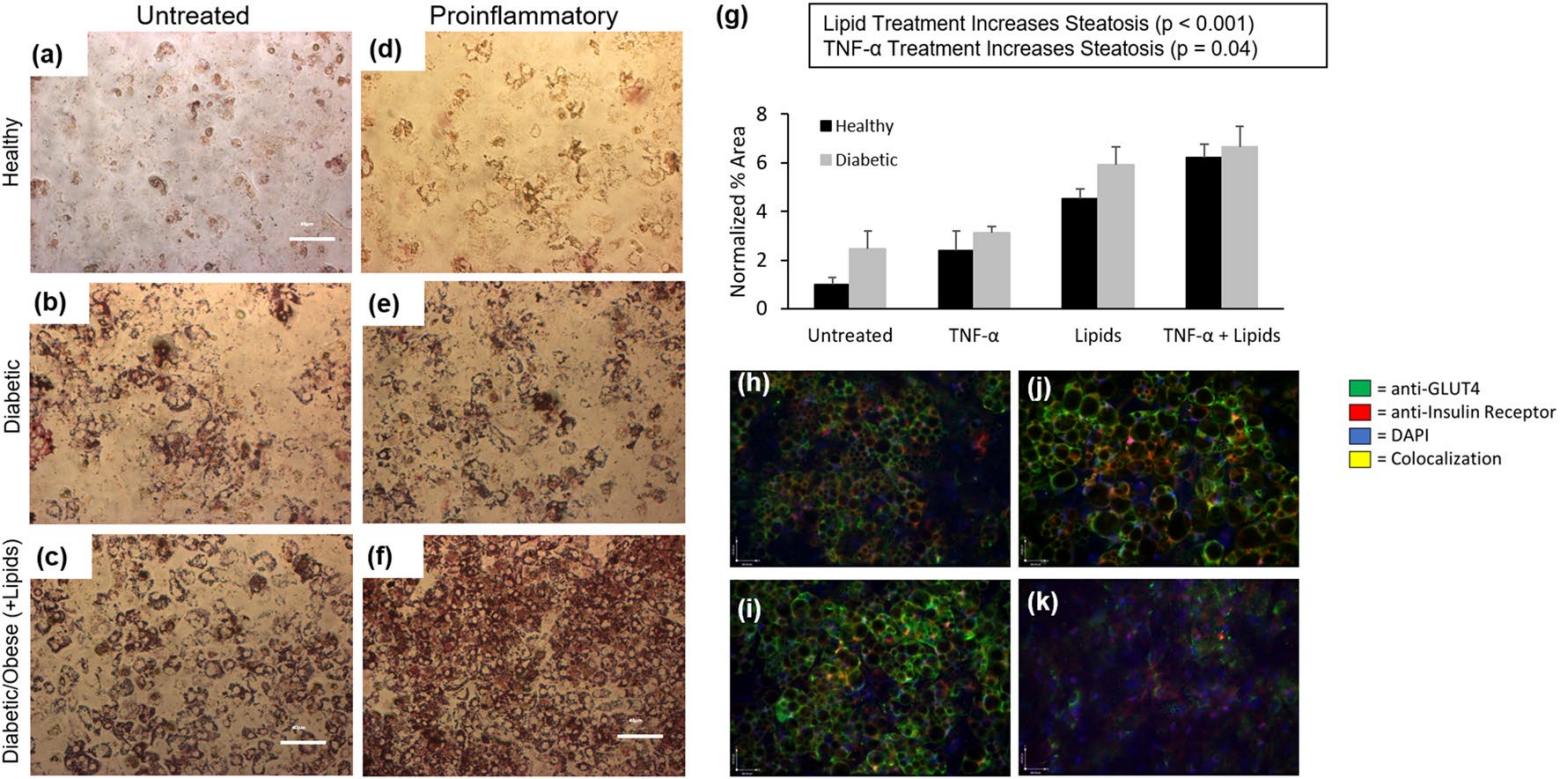
Expression of NAFLD phenotype in 2-chamber systems. Oil Red O staining showing lipid accumulation in hepatocytes after 14 days in recirculating 2-chamber systems containing (**a**) Healthy BMM (**b**) Diabetic BMM (**c**) Diabetic + obese BMM (**d**) Proinflammatory BMM (**e**) Diabetic + proinflammatory BMM (**f**) Diabetic, obese + proinflammatory BMM). (**g**) Quantification of hepatocyte lipid accumulation. ICC quantification of insulin resistance biomarkers after 14d in recirculating (**h**) Healthy BMM (**i**) Diabetic BMM (**j**) Diabetic + Obese BMM (**k**) Diabetic, obese, and proinflammatory BMM. Statistical analysis performed as multi-way ANOVA followed by Dunnett’s test (α = 0.05) using the coverslip-wide average of two independent coverslips per condition.

Steatosis was analyzed as an endpoint readout for hepatocytes (liver module) after co-culture with adipocytes in a 2-chamber platform (A–L system). While TNF-α treatment did not drive increased hepatocyte steatosis in monoculture, both obese (*p* < 0.001) and proinflammatory (*p* = 0.04) medium formulations increased hepatocyte steatosis, in the A–L system with adipocytes as shown in Fig. 4. Compared to untreated hepatocytes in healthy medium (Fig. 4A), steatosis increased in both healthy (Fig. 4D) and diabetic (Fig. 4E) medium for TNF-α treated cells. Lipid treatment also drove an increase in steatosis in both healthy and diabetic media. Steatosis increases were statistically significant for both obese (*p* < 0.001) and proinflammatory media (*p* = 0.04). The highest level of steatosis observed was for the combination of proinflammatory containing obese medium, increasing relative to the obese medium alone by 6.2 × in healthy and 6.6 × in diabetic medium compared to the healthy control (Fig. 4G). This is consistent with the role of TNF-α in vivo, where it is secreted primarily by macrophages that infiltrate adipose tissue, driving adipocyte insulin resistance and lipolysis, decreased efficiency of lipid storage in white adipose tissue and increased serum free fatty acids, which are stored ectopically as triglycerides in the liver^31,32^.

### Hepatocytes show altered CYP3A4 activity in proinflammatory medium

**Figure 5:**
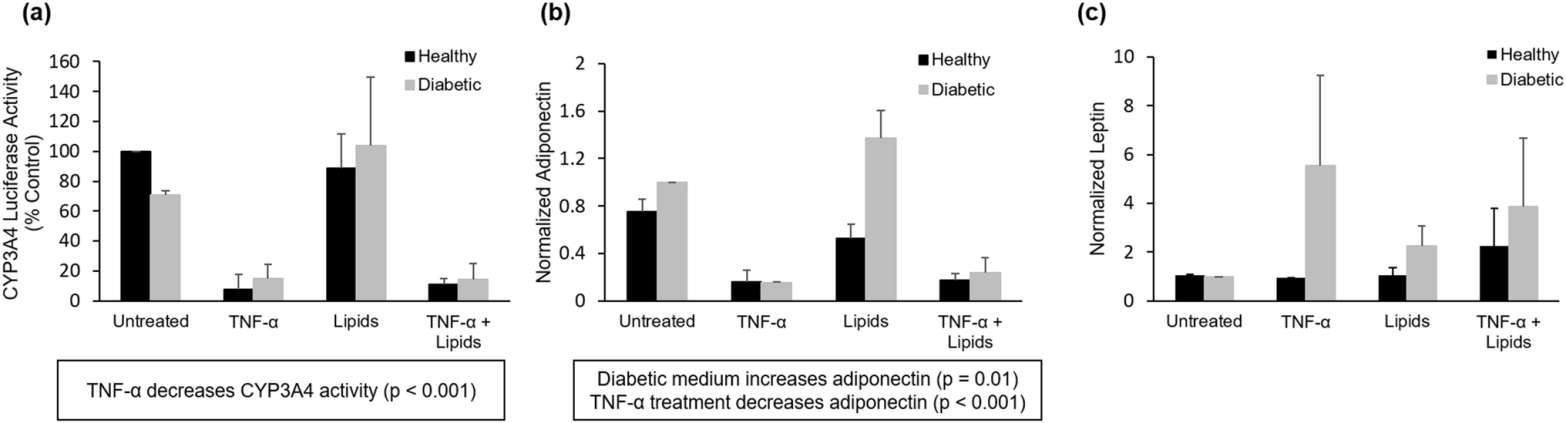
Cell function and biomolecule detection. (**a**) Viability-normalized hepatic CYP3A4 activity after 14d in recirculating systems. (**b**) Adipocyte adiponectin secretion (**c**) Adipocyte leptin secretion. Statistical analysis performed as multi-way ANOVA followed by Dunnett’s test (α = 0.05) using two independent coverslips per condition.

Hepatocyte function in the A–L system was assessed through the measurement of cytochrome P450 enzymatic activity (Fig. 5A). Hepatocytes in the A–L system exhibited decreased CYP3A4 activity in proinflammatory medium and in proinflammatory/obese medium when normalized for viability (*p* < 0.001). This is consistent with in vivo studies where CYP3A4 mRNA expression is found to be lowered in NASH patients, and enzymatic activity is lowered in NAFLD in vitro models^33^.

### Adipocytes show altered adipokine secretion in proinflammatory medium

To analyze secretion of adipokines from adipocytes, soluble leptin and adiponectin from the medium were analyzed at a timepoint between day 6 and 9 in A–L systems. It has been established that leptin and adiponectin ratios are altered in obesity, and adiponectin is associated with protection against insulin resistance in hepatocytes and visceral adipocytes, and adiponectin levels are predictive of NAFLD^34^. In both monoculture (data not shown) and A–L systems, TNF-α treatment decreased adiponectin secretion (Fig. 5B) (*p* < 0.001) while leptin secretion (Fig. 5C) was maintained or increased (though no statistically significant increase in leptin secretion). Compared to untreated cells in healthy medium, the medium of cells treated with TNF-α exhibited decreases in soluble adiponectin, alone and with the combination of lipid addition. No significant changes in adiponectin secretion were observed with diabetic or lipid-treated medium. Leptin secreted in diabetic plus proinflammatory conditions with and without lipids indicated a slight increase, establishing that secretion changes represent a change in adiponectin to leptin ratios and not a total decrease in functionality in diseased conditions.

### The insulin-sensitizing drug metformin significantly affects steatosis at higher than physiological doses

**Figure 6:**
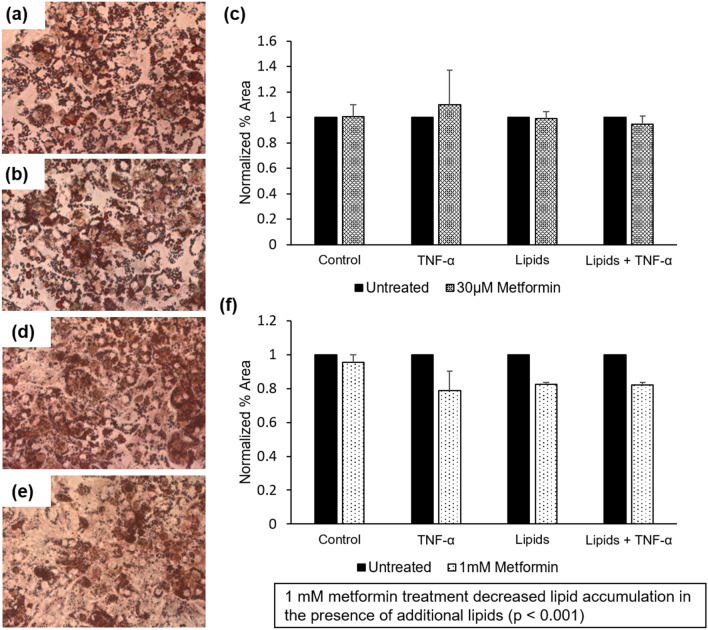
Oil Red O staining showing lipid accumulation in hepatocytes after 14 days in recirculating diabetic, obese, proinflammatory BMM with (**a**) Control (**b**) 30 μM Metformin. (**c**) Quantification of effect of metformin for each medium condition. Oil Red O staining showing lipid accumulation in hepatocytes after 7 days in recirculating diabetic, obese, proinflammatory BMM with (**d**) Control (**e**) 1 mM Metformin. (**f**) Quantification of the effect of metformin for each medium condition. Statistical analysis was performed with a one-way ANOVA followed by Dunnet’s test with the relative change in each condition to block for TNF-α addition and lipid addition (α = 0.05) using between 2 and 4 independent coverslips (**e**) or 3 independent coverslips per condition (**f**).

The mechanism of action of metformin is not fully understood, but the drug has been established to decrease *de* novo glucose production in the liver, decrease hepatic fatty acid synthesis, and increase the insulin sensitivity of multiple tissues including the liver, adipose tissue, and skeletal muscle^35^. To assess the effects of metformin on steatosis, A–L systems were treated with metformin at a dose of 30 μM for 14 days. This value corresponds to the expected peak serum concentration of the drug in dosed patients^36^, though the overall concentration in vivo can vary from 1 to 50 μM with higher doses at the portal tract and accumulation in gastrointestinal tissues^37^. With systemic dosing, hepatocytes did not show a statistically significant decrease in steatosis in any diabetic medium formulation (Fig. 6A–C) (*p* = 0.9).

To explore the effects of a higher, acute dose of metformin in our model, a 1 mM metformin dose was used in a shortened 7-day treatment. Increases in metformin concentration led to hepatocyte toxicity at doses above 1 mM, and cell death was observed before day 14 at the 1 mM dose (data not shown). With this protocol, a decrease in steatosis was observed compared to the control conditions among all diabetic medium formulations (Fig. 6D–F) (*p* < 0.001). While metformin treatment did not ameliorate steatosis entirely, a significant decrease was observed in the lipid-containing medium formulations; 1 mM metformin decreased diabetic/obese condition steatosis to 82% of the control and diabetic/obese/proinflammatory condition steatosis to 82.1% of the control. These results model the discrepancies found in drug treatment results in vivo compared to preclinical prediction; while metformin can decrease hepatocyte steatosis in doses above physiological range in isolated hepatocytes, these results do not translate to an effective treatment for steatosis.

## Discussion

We have developed the first human-based multi-chamber HoaC model comprised of both hepatocytes and white adipocytes that enabled the modeling of NAFLD phenotypes in both liver and adipose modules as well as organ cross-talk. This model allowed the exploration of the role of adipocyte lipolysis and insulin resistance in NAFLD, as well as the exchange of cytokines and adipokines between organs. In addition, it evaluated the discrepancy between preclinical animal response to metformin and clinical treatment.

Because of fundamental differences between humans and rodents, animal models for NAFLD tend to rely on genetic metabolic dysfunction to model an NAFLD phenotype. This human-based HoaC model allowed the study of the metabolic element of NAFLD by mimicking critical aspects of the in vivo conditions for diabetes, obesity, and low-grade adipose tissue inflammation. The proinflammatory media models elevated TNF-α, which is produced normally by adipocytes, but is highly elevated through the influence of macrophages that infiltrate adipose tissue in metabolic disease, T2D, and NAFLD. While the macrophage population in adipose tissue is approximately 5% by cell number in lean individuals, in obesity, the numbers can rise as high as 50% macrophages in WAT^38^. TNF-α elevation correlates with adipocyte dysfunction, lipolysis, and insulin resistance and is thought to play a role in systemic insulin resistance^39^. In these studies, TNF-α is only sufficient to increase hepatocyte steatosis in the presence of adipocytes, suggesting that TNF-α does not cause a direct increase in liver steatosis, but acts through modulation of adipokine secretion profiles and adipose lipolysis. This is supported by an increase in insulin resistance markers and a decrease in adiponectin secretion in the adipose module of the multi-organ A–L system. This result is consistent with clinical studies that indicated low-grade adipose tissue inflammation is a major driving force for NAFLD, and that adipose tissue insulin resistance is an initiating factor in disease^40^. Further, this model shows evidence of a phenotype beyond simple steatosis toward an advancement to NASH; proinflammatory medium and adipocytes in the A–L system drove a decrease in function as shown by decreased CYP3A4 activity. NAFLD-associated proinflammatory molecules and oxidative stress downregulate expression and activity of key drug-metabolizing enzymes and transporters, including enzymes in the CYP3A family, the activity of which decreases with steatosis severity and NAFLD progression. Alterations in CYP3A4, which plays a role in the metabolism for about half of clinically applicable drugs, has the potential to drive variable drug response in patients with NAFLD and NASH, further complicating treatment strategies for patients with metabolic disease^41^.

We have also shown that this platform displays physiologically relevant responses to pharmaceutical treatments. Metformin altered hepatic steatosis only at concentrations above those typically used in humans; while increased concentrations decreased steatosis, hepatocyte toxicity issues prevented dosing for a full 14 days. These results reflect the fact that other researchers testing for metformin efficacy typically performed acute, 24- or 48-h assays at high concentrations, which would allow for detection of changes in lipid storage without significant cell death, but not predict longer term in vivo results which are run at much lower concentrations. However, because efficacy in human clinical treatment is low for metformin, improvements in steatosis in vitro may not be considered a physiologically relevant response. These discrepancies highlight the need for chronic dosing studies using representative human cells and models that recapitulate relationships between relevant tissues in addition to individual cell response.

The microfluidic flow across the hepatocytes induces a shear stress condition on the cells in the device, which is known to induce changes in the hepatocytes, increasing in vivo-like hepatocyte function^42^. The shear stress in this microfluidic system is sinusoidal and bidirectional, which may have a more complex influence on the hepatocytes than continuous linear flow. This platform could be used to further investigate shear stress and flow profiles through alteration of flow paths and rocking profiles to both study this influence on disease progression as well as to increase the physiological similarity in the system to in vivo. Another alteration for future work includes the addition of liver sinusoidal endothelial cells (LSECs), which have a major contribution to the progression of many liver diseases, affect both stellate and non-stellate liver cells, and are responsive to shear stress^43^. The complex interaction of the LSECs with the recirculating medium and the stellate cells and hepatocytes could be added to the system and studied in a number of configurations, including as a barrier, in physical contact with the hepatocytes and stellate cells, or physically separated but in communication via soluble factors transported in the recirculating flow system.

Significant effort has been put into the development of a pharmaceutical treatment for NAFLD; while T2D medications like metformin can be helpful for metabolic syndrome, where full-body insulin resistance contributes to overall disease, no drug on the market is aimed at treating NAFLD or NASH directly. This highlights the need for more representative platforms for studying the disease and for evaluating new pharmaceuticals. While a 2D cell layer is limited in its ability to model complex liver architecture and does not include other cell types relevant to NAFLD including stellate cells, sinusoidal endothelial cells, and Kupffer cells, a platform with recirculating serum-free medium utilizing gravity-based flow allows for a low-volume platform that facilitates integration with other inter-connected modules and allows cellular cross talk at physiologically relevant concentrations. This work validates a new human-on-a-chip platform which can model adipocyte-hepatocyte interactions, give insight into drug response, and shows potential for the testing of novel compounds aimed at treating NAFLD and NASH in a human-based model.

## Methods

### System fabrication

The two-chamber housing system was designed using computational fluid dynamics (CFD) with CFD-ACE + to establish viable fluid flow rates and shear stresses and Autodesk AutoCAD, using poly(methyl methacrylate) housings and poly(dimethyl siloxane) gaskets. Both the poly(methyl methacrylate) and poly(dimethyl siloxane) components were laser cut using a Trotec Speedy 400 CO_2_ laser cutter. System design included two circular regions sized for 15 mm coverslips containing the cellular components, two 10 mm diameter reservoirs used to facilitate gravity-driven recirculating flow, and flow paths among these four compartments.

### Cell culture

Cryopreserved primary human liver cells were obtained from Massachusetts General Hospital (MGH, Boston, MA, USA, lot#HW54). Cells were plated after thawing on ECL Cell Attachment Matrix (Sigma) on 70% isopropanol-sterilized 15 mm glass coverslips (cs), at a density of approximately 250,000 cells/cs. Cells were cultured in vendor-recommended medium^28^ for 5–9 days before incorporation into systems. Medium was replaced fully every 48 h as previously described^25,28^.

Primary human cardiac preadipocytes (Cell Applications, Sigma) were plated and began differentiation into mature adipocytes approximately 2 weeks before incorporation into systems. Cells were plated onto 70% isopropanol-sterilized 15 mm glass coverslips at a density of 90,000 cells/cs in Preadipocyte Growth Medium (Cell Applications, Sigma). Preadipocytes were cultured in growth medium for 1–3 days until confluent, and medium was replaced with Adipocyte Differentiation Medium (Cell Applications, Sigma). Differentiation of cells to mature adipocytes was designated when cells exhibited extensive lipid droplet accumulation.

For experiments in monoculture, treatment was performed in-plate, and cells remained in plate for testing/readouts. Prior to functional or endpoint assays, two-organ systems were disassembled and the organ modules were transferred to well-plates.

### Medium preparation

During all experiments, monocultures and systems were maintained in their respective metabolic medium. The base serum-free, blood-mimetic medium (BMM) was formulated as previously described^25^, with modification in glucose and insulin. The base medium was replaced with 1X Neurobasal-A medium, no D-glucose, no sodium pyruvate (Life Technologies, A24775-01) and 1X B27 minus insulin (Life Technologies, A1895601), and was supplemented with 0.22 mM sodium pyruvate (Sigma, S8636).

To rudimentarily mimic the healthy and disease blood plasma conditions relevant to NAFLD and NASH, four different media formulations were prepared. The healthy condition was mimicked by supplementing the base medium with post-prandial glucose (5 mM) and insulin (1 nM) concentrations (Healthy BMM), and the diabetic condition was mimicked by supplementing the base medium with 25 mM glucose and 69 μM insulin, concentrations within the range of a post-prandial diabetic patient (Diabetic BMM)^44^. The proinflammatory plasma condition was mimicked by supplementing healthy BMM with 10 μM tumor necrosis factor alpha (TNF-α) (Sigma, T0157)^45^. The obese plasma condition was mimicked by supplementing healthy BMM with physiologically-relevant concentrations of free fatty acids: 45 μM BSA-conjugated palmitate (Sigma, A8806) and 65 μM oleate (Sigma, O3880) using stocks prepared according to the manufacturer. Combinatorial treatment conditions were prepared by combining the individual diseases formulations described above.

### Multi-organ experimental procedure

Adipose and hepatocyte coverslips were transferred cell-side up to chambers in the bottom housing and covered with Healthy BMM. After securing coverslips in housings, systems were filled with medium and incubated at 37 °C on a rocker following a sinusoidal profile of 1° tilt amplitude and period of 60 s. After 24 h, medium was replaced with treatment condition medium. Cells were viewed and imaged daily on a phase microscope, and a 300μL medium change was performed every 48 h. After 14 days of treatment, systems were disassembled, and cell coverslips were transferred to separate plates for downstream analysis.

### Flow cytometry

For analysis of differentiation markers in adipocytes, after differentiation in monoculture, cells were disassociated using 0.25% trypsin, centrifuged at 300G, and resuspended in FACS buffer consisting of 1% BSA (Sigma) in PBS. Samples were treated with Human FcR Block (Miltenyi Biotec) before analysis. Cytometer analysis was used to detect expression of FABP4 (Abcam, ab93945), CD200 (Abcam, ab134489), and APC/750 for live/dead analysis and as a control.

### Immunocytochemistry

For analysis of insulin receptor and GLUT4 expression, adipocytes were fixed with 4% paraformaldehyde (PFA) for 10 min and rinsed 2 × with 1 × PBS. Nonspecific binding sites were blocked for 60 min (1% BSA, 5% goat serum in 1X PBS). Cells were incubated with primary antibodies (1:150 rabbit anti-α-insulin receptor antibody, β subunit (Sigma, 07–724), 1:150 mouse anti-GLUT4 antibody (Invitrogen, MA5-17176)) overnight in the dark, at 4 °C. The following day, primary solution was removed, and cells were washed 3X with 1X PBS. Cells were treated with secondary antibodies (1:200 dilution Donkey anti-Mouse IgG Alexa Fluor 488 (Invitrogen A21202), Goat anti-Rabbit IgG Alexa Fluor 568 (Invitrogen, A11036)) for 2 h in the dark, washed 2 × with PBS and DNA stained using 3 mM 4′,6-diamidino-2-phenylindole (DAPI) reagent for 5 min. Cells were washed 2 × with PBS and mounted on slides with ProLong Diamond Antifade Mountant (Invitrogen, P36970).

### Alamar blue assay

Following system disassembly, cells were treated with alamarBlue reagent (Bio-Rad BUF012A) diluted to 10% in healthy BMM. Coverslips were incubated 4–8 h until a color shift was observed, and a Bio-Tek synergy HT Microplate Reader was used to measure fluorescence using an excitation wavelength of 560 nm and an emission wavelength of 590 nm.

### CYP 3A4 assay

On day 14 of treatment, enzymatic activity was assessed (P450-GloCYP3A4 Assay and Screening System, Promega). Briefly, systems were disassembled, and the hepatocyte coverslips removed and incubated in DMEM with no phenol red (Invitrogen, A1443001) for 60 min with luciferin-IPA precursor solution. Supernatant was reserved and frozen at -80 °C until analysis. A secondary reaction with P450-Glow reagent was performed to generate oxyluciferin. Luminescence was measured using a Bio-Tek synergy HT Microplate Reader.

### Detection of leptin and adiponectin

Soluble leptin and adiponectin were detected in reserved 2-chamber system medium, or adipocyte medium from monoculture experiments. Frozen samples were thawed and centrifuged before the assay was performed. Leptin was analyzed using a Human Leptin ELISA Kit following the manufacturer’s protocol (Abcam, ab179884). Adiponectin was analyzed using Human Adiponectin ELISA Kit following the manufacturer’s protocol (Abcam, ab99968).

### Oil Red O analysis

For analysis of stored lipids in hepatocytes, following CYP analysis as described above, cells were analyzed using Oil Red O, a lipid-soluble diazo dye that gives lipid-storing vesicles a red color as an endpoint readout. Cells were fixed in 4% PFA for 10 min and rinsed twice with PBS. Coverslips were treated with an Oil Red O suspension (3:2 ratio of Oil Red O dye (0.5% in isopropanol, Sigma) to sterile water) for 30 min on an orbital shaker. Excess dye was removed, and coverslips were rinsed 2 times with PBS. Cells were imaged at 20 × magnification using phase microscopy. Steatosis was analyzed using ImageJ Fiji software^46^; images were binarized and gated for intensity to isolate dark red lipid droplets, and particles were separated using Watershed separation^47^. A percentage area of signal gated to be lipid storage was generated for each image. A minimum of 3 images per coverslip were analyzed and averaged.

### Statistical analysis

Data was statistically compared using SigmaPlot using multi-way ANOVA followed by Dunnett’s test (α = 0.05). Specifically, a three-way ANOVA with factors of Healthy vs Diabetic medium, TNF-α addition, and lipid addition, with Dunnett’s test was used to compare to the healthy, no TNF-α, and no lipid addition conditions for the following evaluations: adipocyte lipid accumulation (both in monoculture and two-chamber systems), CYP3A4 activity, adiponectin, and leptin. The effect of metformin on lipid accumulation was performed with a one-way ANOVA followed by Dunnett’s test with the relative change in each condition to block for TNF-α addition and lipid addition.

## Data Availability

Data supplied upon request to the corresponding author.
